# Comparative Analysis of Apicoplast-Targeted Protein Extension Lengths in Apicomplexan Parasites

**DOI:** 10.1155/2015/452958

**Published:** 2015-05-31

**Authors:** Alexandr V. Seliverstov, Oleg A. Zverkov, Svetlana N. Istomina, Sergey A. Pirogov, Philip S. Kitsis

**Affiliations:** Institute for Information Transmission Problems of the Russian Academy of Sciences (Kharkevich Institute), Bolshoy Karetny Pereulok 19, Moscow 127994, Russia

## Abstract

In general, the mechanism of protein translocation through the apicoplast membrane requires a specific extension of a functionally important region of the apicoplast-targeted proteins. The corresponding signal peptides were detected in many apicomplexans but not in the majority of apicoplast-targeted proteins in *Toxoplasma gondii*. In *T. gondii* signal peptides are either much diverged or their extension region is processed, which in either case makes the situation different from other studied apicomplexans. We propose a statistic method to compare extensions of the functionally important regions of apicoplast-targeted proteins. More specifically, we provide a comparison of extension lengths of orthologous apicoplast-targeted proteins in apicomplexan parasites. We focus on results obtained for the model species *T. gondii*, *Neospora caninum*, and *Plasmodium falciparum*. With our method, cross species comparisons demonstrate that, in average, apicoplast-targeted protein extensions in *T. gondii* are 1.5-fold longer than in *N. caninum* and 2-fold longer than in *P. falciparum*. Extensions in *P. falciparum* less than 87 residues in size are longer than the corresponding extensions in *N. caninum* and, reversely, are shorter if they exceed 88 residues.

## 1. Introduction

In general, the mechanism of protein translocation through the apicoplast membrane requires a specific extension of a functionally important region of the apicoplast-targeted proteins. In* T. gondii* signal peptides are either much diverged or their extension region is processed, which in either case makes the situation different from other studied apicomplexans. We propose a statistic method to compare extensions of the functionally important regions of apicoplast-targeted proteins. More specifically, we provide a comparison of extension lengths of orthologous apicoplast-targeted proteins in apicomplexan parasites. We ground on the notion that the majority of cyanobacterial proteins lack such extensions (including signal peptides) and consist of only functional sequences.

Sporozoans comprise a monophyletic lineage of apicomplexan parasites. Among them,* Toxoplasma gondii* is an important medical and veterinary pathogen commonly causing morbidity in HIV patients [1, 2]. The study [3, 4] describes the propagation mechanism of* T. gondii* in various hosts worldwide, including several aquatic mammal species, where it may provoke abortion and lethal systemic disease. The observation that the* apicoplast* of* T. gondii* significantly varies in shape and protein expression patterns at different life stages of the parasite suggests its important role in virulence; the apicoplast of* T. gondii* is also involved in the pathogen stage conversion and the parasite proliferation [5]. Due to bacterial origin of the apicoplast proteins, they present a natural target for selective treatment in the eukaryotic host. The sporozoans contain a semiautonomous organelle, the apicoplast, acquired by secondary endosymbiosis with ancient red algae; plastid organelles of the red algae originate from cyanobacteria [6–8].

Elucidating the molecular mechanism that underlies the role of apicoplast in the parasite invasion, conversion, and proliferation is important for development of novel therapeutics to control infection and reactivation of the parasite. Further analysis of unique features of apicoplast-targeted proteins (particularly, regions involved in translocation processes) in* T. gondii* can add to the effective design of drug-based or genetic strategies to control the pathogen development and proliferation. At the reported stage of the study, we analyze only extensions in length of orthologs among apicomplexan parasites.

Note that the coccidian* Cryptosporidium parvum* lacks the apicoplast [9], and the apicoplast in piroplasmids* Babesia bovis* and* Theileria parva* largely differs from that in common coccidians and the haemosporidian* Plasmodium* spp. [10, 11].

The majority of apicoplast proteins are encoded in the nucleus and only few in its own genome. Most of these proteins can be identified due to their cyanobacterial origin. Transport of nuclear-encoded proteins to the apicoplast in* T. gondii* is significantly less documented experimentally compared to* Plasmodium falciparum*. Among the documented cases is the nuclear-encoded lipoic acid synthetase LipA [12]; other examples are described in [13–15]. A mechanism of protein import into secondary plastids is also described in [16], where many orthologous proteins involved in this process were shown to be presented in the sporozoans* P. falciparum* and* T. gondii*, cryptophyte alga* Guillardia theta*, and diatom* Phaeodactylum tricornutum*. Plastids also possess the bacterial system to translocate folded proteins [17, 18].

A variety of protein localization prediction methods are used to identify apicoplast-targeted proteins. Some of them utilize the notion that translocation across the four membranes surrounding the apicoplast is mediated by an N-terminal bipartite targeting sequence, a special N-terminal signal, and a transit peptide [13]. The algorithm ApicoAP described in [19] predicts apicoplast-targeted proteins containing the signal peptide, because it trains on a learning sample of signal peptide-containing proteins. Other apicoplast-targeted proteins are predicted neither with this algorithm nor with ApicoAMP [20]. The comprehensive ToxoDB database constructed using the SignalP algorithm contains proteins with the information on presence/absence of the signal peptide. According to this database, some nuclear-encoded proteins in* T. gondii* that are experimentally shown to reach the apicoplast should do not contain signal peptides, albeit bearing housekeeping functions in the apicoplast. The methods in application to* Plasmodium* spp. are described, for example, in [19, 21–23]. The PlasmoAP algorithm [24] is designed specifically for* Plasmodium* spp. and is of little applicability to coccidians. Hence, these two widely used databases may be considered of limited use to identify apicoplast-targeted proteins not containing the standard signal peptide in coccidians. We therefore applied a crude technique to compare apicomplexan proteins with their orthologs in a cyanobacterium. Namely, orthology between nuclear-encoded sporozoan proteins and cyanobacterial proteins is used as a basis to suggest the apicoplast-targeted nature of the proteins. As our study relies on statistic estimates, its predictions are hopefully not affected by the chosen parameters of global protein alignment.

In this work, the lengths of sporozoan proteins are compared with each other and with the length of their orthologs in the cyanobacterium* Synechocystis* sp. PCC 6803. We consider lengths of the sporozoan proteins that extend outside the conserved alignment region, which usually covers the entire cyanobacterial sequence. We focus on results obtained for the model species* T. gondii*,* Neospora caninum* (the two coccidian sporozoans with completed genome projects, as per the end of 2013), and the malaria agent* P. falciparum* from the Haemosporidia.

Based on the total comparison, we conclude that* T. gondii* in most cases contains longer proteins compared to both* N. caninum* and* P. falciparum*. We also surmised that at least some of them undergo processing in the cytoplasm to facilitate transporting into the apicoplast. The extended portions of proteins may also be involved in gene expression regulation at the level of protein-protein interaction.

As an argument, the regulation of plastid-encoded genes* ycf24* and* rps*4 affects the general functionality of the apicoplast in* T. gondii* [25]. The expression regulation of* ycf24* (the SufB factor mediating the Fe-S cluster assembly in many nuclear proteins) was suggested to take place in the apicomplexans* Eimeria tenella*,* T. gondii* RH, and* Plasmodium* spp., as well as in* Gracilaria tenuistipitata*,* Porphyra purpurea*, and* Porphyra yezoensis* [25]. The same type of regulation was suggested for* rps*4 (ribosomal protein S4) in* T. gondii* RH [25].

## 2. Materials and Methods

Protein data for* T. gondii* and* N. caninum* was extracted from the ToxoDB database (version 8.2), data for* Plasmodium* spp. from the PlasmoDB database (version 9.3), and data for* Synechocystis* sp. PCC 6803 from GenBank, NCBI [26]. ToxoDB and PlasmoDB are specialized, regularly updated, and nonoverlapping databases. Conserved domains were detected according to the Pfam database [27]. The location of regions enriched with a certain amino acid was established using the PROSITE database [28].

We compared the proteomes of three apicomplexan parasites (*T. gondii* ME49,* N. caninum* Liverpool, and* P. falciparum* 3D7) and the cyanobacterium* Synechocystis* sp. PCC 6803. For each pair of proteomes, pairs of orthologous proteins were computed on the basis of an alignment quality score using the Needleman-Wunsch method and BLOSUM62 matrix [29, 30].

Our method to study the lengths of the apicomplexan protein extensions is as follows. For each cyanobacterial protein with length *s* and its two orthologs (from a fixed pair of apicomplexans) with lengths *a* and *a*′, the point with coordinates (*a* − *s*, *a*′ − *s*) is computed. In some cases, one or both of the coordinates are negative, which indicates a sporadic case of a shorter length of the sporozoan protein versus the cyanobacteria.

**Figure 1:**
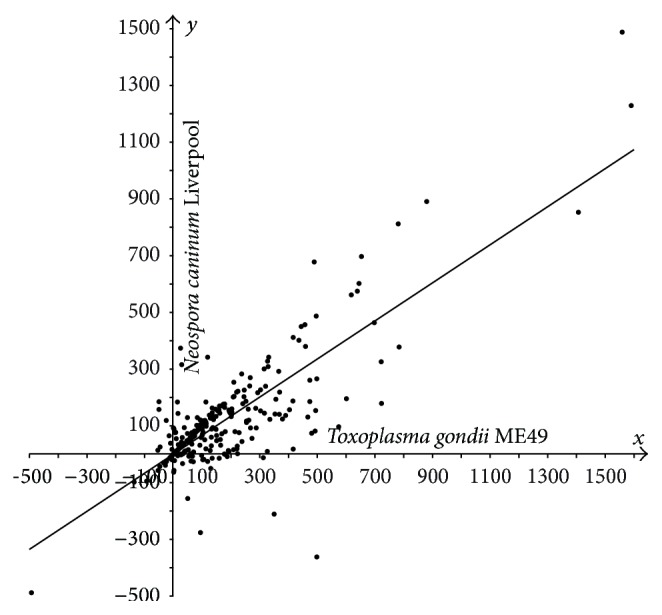
Plot of protein length extensions in* T. gondii* ME49 versus* N. caninum* relative to their orthologs in* Synechocystis* sp. PCC 6803. Differences in lengths between sporozoan and cyanobacterial orthologous proteins are plotted as follows:* T*-*S* on the *x*-axis,* N*-*S* on the *y*-axis.

**Figure 2:**
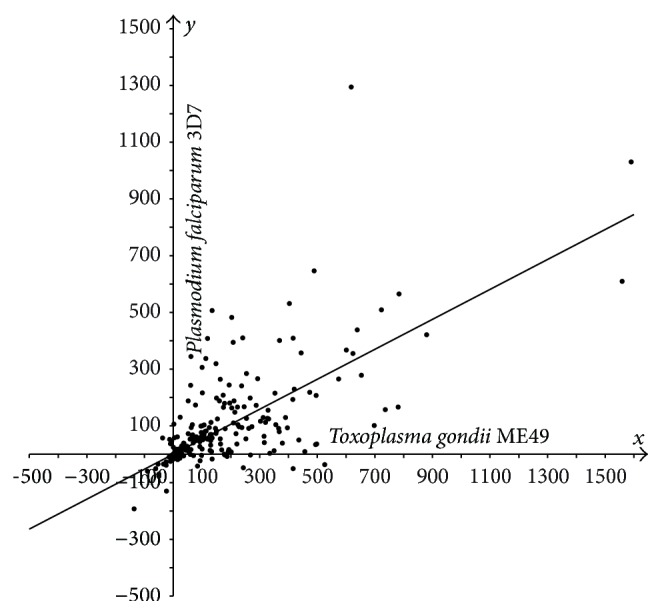
Plot of protein length extensions in* T. gondii* ME49 versus* P. falciparum* 3D7 relative to their orthologs in* Synechocystis* sp. PCC 6803. Differences in lengths between sporozoan and cyanobacterial orthologous proteins are plotted as follows:* T*-*S* on the *x*-axis,* P*-*S* on the *y*-axis.

**Figure 3:**
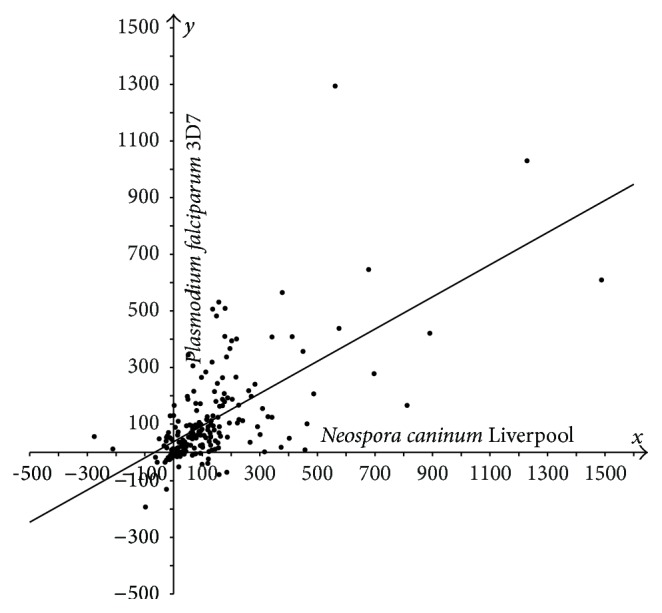
Plot of protein length extensions in* N. caninum* Liverpool versus* P. falciparum* 3D7 relative to their orthologs in* Synechocystis* sp. PCC 6803. Differences in lengths between sporozoan and cyanobacterial orthologous proteins are plotted as follows:* N*-*S* on the *x*-axis,* P*-*S* on the *y*-axis.

In Figures 1–3, the cases of* N. caninum*-*T. gondii* (*N-T*),* P. falciparum*-*T. gondii* (*P-T*), and* P. falciparum*-*N. caninum* (*P-N*) are analyzed using three sets of points. Each coordinate is the difference in lengths between the sporozoan and cyanobacterial orthologs:* T. gondii* versus* Synechocystis* (*T-S*),* N. caninum* versus* Synechocystis* (*N-S*), and* P. falciparum* versus* Synechocystis* (*P-S*). The sets of points are then statistically analyzed. The following statistic was used to test the hypotheses that “a constant is better compatible with the set of points {(*x*_*i*_, *y*_*i*_)∣1 ≤ *i* ≤ *n*} than the nontrivial affine function *y* = *kx* + *b*, *k* ≠ 0” and “the linear function *y* = *kx* is better compatible with this set of points than the affine function *y* = *kx* + *b*, *b* ≠ 0”: $F=\sqrt{\left(\left(\sum_{i}^{}(y_{i}-{{\hat{y}}_{0}}_{}(x_{i}){)}^{2}/\sum_{i}^{}(y_{i}-\hat{y}(x_{i}){)}^{2}\right)-1\right)\cdot (n-2)}$, where ${\hat{y}}_{0}$ in the numerator is a constant (mean $\overset{-}{y}$ over all *y*) or linear regression *y* = *kx* and $\hat{y}$ in the denominator is affine regression *y* = *kx* + *b*. This statistic can be explained more clearly: it determines whether there is a correlation between the difference $y_{i}-\hat{y}(x_{i})$ and *x*_*i*_. This statistic is standard and substantiated in [31, 32]. The value of *F* was compared against a threshold defined as the Student random variable at significance level *α*, *t*(*n* − 2, *α*). Under the number of degrees of freedom *n* − 2 > 30, the Student and standard Gaussian distributions approximate each other, and the threshold *t*(266,0.05) equals 1.96. An analogous statistic was used to test the hypothesis “affine function versus general polynomial of second degree.” The confidence interval radius and the radius of the intercept (further referred to as* radius*) for the affine regression slope as well as the slope coefficient radius for linear regression were calculated in a standard fashion [32]. The Student test statistic *S* was used as well [32]. Deming regression and screening singular points were tested as well.

**Figure 4:**
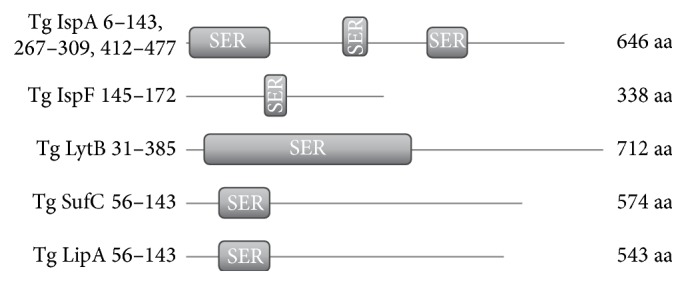
Some apicoplast-targeted proteins in* T. gondii* containing serine-rich regions. The region coordinates indicated on the left and the protein lengths on the right. The serine-rich regions are arranged irregularly, which is confirmed on a larger number of proteins for different pairs, triples, and so forth of amino acids.

Regions of proteins with a predominance of one amino acid were determined by using the PROSITE program. The distribution of amino acid pairs separated by a fixed distance *k* in a given set of amino acid sequences was established using the simple computer program available from http://lab6.iitp.ru/utils/aapf/. Namely, frequencies of all amino acid pairs occurring in the given sequences at the distance of *k* residues (specified in the interval from 0 to 255) are computed and averaged over all sequences. The output is a frequency matrix of amino acid pairs. This matrix can be used to characterize nonstandard types of the putative signal peptide. This way it also appeared impossible to determine specificity of the N-terminus of apicoplast-targeted proteins in* T. gondii*; refer to Figure 4.

## 3. Results and Discussion

Orthologs of* Synechocystis* sp. PCC 6803 were identified for 515 of 8319 (~6%) nuclear proteins in* T. gondii*, 560 of 7122 (~8%) nuclear proteins in* N. caninum*, and 390 of 5538 (~7%) nuclear proteins in* P. falciparum*. Only 877 of 3179 (~28%) proteins in* Synechocystis* sp. were found to be orthologous against at least one of the three apicomplexan species (see Supplementary Material available online at http://dx.doi.org/10.1155/2015/452958).

The identified orthologs are putative apicoplast-targeted proteins. Among them are proteins with either experimentally shown or anticipated apicoplast affinity, such as the bacterial type RNA polymerase sigma subunit (RpoD), DNA ligase, aminoacyl-tRNA synthetases, cell-cycle-associated protein kinase PRP4, enzymes IspA, IspB, IspE, IspF, IspG (GpcE), and IspH (LytB) of the mevalonate-independent pathway of isoprenoid biosynthesis, sulphur mobilization protein SufC from a Fe-S cluster assembly pathway, and LipA and LipB enzymes of lipoic acid synthesis (refer to the Introduction [5, 12, 14, 15]). In pairwise alignments, the sporozoan and cyanobacterial proteins usually align well at their C-termini, and the cyanobacterial sequence is fully covered by the alignment. In many cases, the N-termini of sporozoan proteins extend outside the alignment (data not shown).

In most cases, sporozoan proteins are longer compared to their bacterial orthologs, Figures 1–3. We demonstrate statistically that the majority of proteins in* T. gondii* are considerably longer compared to their orthologs in* N. caninum* Liverpool and* P. falciparum* 3D7, which was evidenced previously only for selected proteins [12, 33].

The hypotheses “a constant is better than the nontrivial affine function” and “affine function versus general polynomial of second degree” were rejected for every three sets of points shown in Figures 1–3. The hypothesis “the linear function is better than the affine function” was compatible with the first two sets (*F* = 0.15 and *F* = 1.57; refer to the designation in Materials and Methods section) and was rejected for the third set (*F* = 3.40). Thus, the third set was tested against the hypothesis “the mean over all* x*-coordinates coincides with the mean over all* y*-coordinates”; this hypothesis was accepted with the Student test statistic *S* at the same significance level *α* (with* S* = 1.547) [32].

Hence, the following regressions were justified. For set 1, *y* = 0.6711*x* with radius 0.0468; for set 2, *y* = 0.528*x* with radius 0.0590; for set 3, *y* = 0.5685*x* + 37.756 (linear regression rejected with *F* = 3.40) with radii 0.0926 and 21.7521, respectively.

The Deming regression gives approximately the same estimates; screening singular points does not significantly affect the results (data not shown).

So, the following conclusions can be drawn for the apicoplast protein orthologs that have orthologs in the cyanobacterium.

1. Protein extensions in* T. gondii* are on average 1.5-fold longer compared to the corresponding extensions in* N. caninum*, with almost 1.0 confidence (Figure 1).
2. Protein extensions in* T. gondii* are on average 2-fold longer compared to the corresponding extensions in* P. falciparum*, with high confidence (Figure 2).
3. Set 3 (Figure 3) is compatible with the hypothesis that the average of protein extension lengths in* N. caninum* equals that in* P. falciparum*. Extension lengths in* P. falciparum* being less than 87 residues are longer than the corresponding extensions in* N. caninum* and, reversely, are shorter if they exceed 88 residues. In units, the dependency between extension lengths in* P. falciparum* versus* N. caninum* is an affine function *y* = 0,5685*x* + 37,756, where *y* runs over extension lengths in* P. falciparum* and *x* in* N. caninum*. The affinity, but not the linearity, of the regression testifies on behalf of the difference of* T. gondii* from her immediate species* P. falciparum* and* N. caninum* once again.

Among other specific features of apicoplast-targeted proteins is the abundance of serine-rich regions revealed in analyses with PROSITE (Figure 4). Each of the 3551 proteins in* T. gondii* ME49 possesses at least one 27 amino acid-long region with at least 9 serine residues, and 39 proteins possess at least one region with 27 or more continuous serine residues. Contrary to our expectations, larger-scale searching for serine-rich motifs in* T. gondii* showed their presence in various protein families, thus suggesting a selectively neutral nature of their origin. In other words, serine-rich regions are not specific to N-termini of apicoplast-targeted proteins. The same is also observed for other amino acids. This approach does not allow detecting a novel type of the N-terminal signal.

Earlier preliminary results are reported in [33].

## 4. Conclusions

For apicomplexan parasites, we suggest a statistically based method to compare the extension lengths of orthologous proteins that have orthologs in the cyanobacterium.

With this method, we demonstrate that the majority of cyanobacterium orthologs in* Toxoplasma gondii* are significantly longer compared to those in both* Neospora caninum* and* Plasmodium falciparum*. These proteins commonly lack signal sequences typical for* Plasmodium* spp. [34]. The corresponding extensions might be essential for regulation of the apicoplast proteins and their translocation into the apicoplast. This notion conforms well with the observation that the apicoplast membrane in* T. gondii* is known to be less permissible, at least against drugs, compared to that in* P. falciparum* (personal communication with Gamaleya Research Institute of Epidemiology and Microbiology). Differences in protein extension lengths between* T. gondii* and other apicomplexan species may suggest different membrane transport mechanisms in these sporozoan groups. Mechanism of regulation and translocation in* T. gondii* may be based on protein processing in the cytoplasm to mature their extended N-termini.

## Supplementary Material

The supplementary table presents a set of orthologs of the cyanobacterium *Synechocystis* sp. PCC 6803 proteins in *Toxoplasma gondii*, *Neospora caninum* and *Plasmodium falciparum* along with the protein lengths.
The first group of columns contains accession numbers of corresponding database entries for *T. gondii*, *N. caninum*, and *P. falciparum* followed by lengths of the proteins. Protein data for *T. gondii* and *N. caninum* is extracted from the ToxoDB database (version 8.2), data for *P. falciparum* – from the PlasmoDB database (version 9.3), data for Synechocystis sp. PCC 6803 – from GenBank, NCBI. The next three columns contain protein annotations (not given for *N. caninum* as being a part of the annotation for *T. gondii*). The next three columns contain the differences T–S, N–S, and P–S. The 354 rows of the Table describe only cyanobacterial proteins having orthologs in at least two sporozoan species.
